# Screening for chronic kidney disease and inequity: Rastreamento de doença renal crônica e iniquidade

**DOI:** 10.1590/1516-3180.2016.0082080516

**Published:** 2016-07-21

**Authors:** Rodrigo Diaz Olmos

**Affiliations:** I MD, PhD. Professor, Department of Internal Medicine, Hospital Universitário (HU), Universidade de São Paulo (USP), São Paulo, SP, Brazil.

To the Editor,

The editorial about screening for chronic kidney disease (CKD) is quite interesting^1^ and gives us a good grasp of the epidemiological burden of this disease and various aspects of its morbidity and mortality, as well as many insights about the complex relations between disease and socioeconomic status. However, the conclusion that screening could potentially reduce inequity in the Brazilian population, based on correlating CKD with socioeconomic status, is flawed.

There is plenty of evidence showing that most screening tends to produce more inequity, rather than reducing it. This is particularly so if it is done in countries where the public health system is insufficiently organized and not strong enough to be regulated in its entirety and/or if screening programs are not publicly organized, thereby leading to so-called “opportunistic screening”.^2^ This is almost always the case in places with an uncoordinated health system and a strong private health sector.

For instance, if screening for CKD were to be started in Brazil, it is certain that within a short period of time, thousands of wealthy low-risk people would undergo this screening and, most probably, the poor low-educated high-risk population would not have the same access to it as enjoyed by the first group.^3^ Apart from this worrisome increase in inequity, introduction of a new screening intervention would also, potentially and paradoxically, increase the degree of harm among those undergoing opportunistic screening precisely because they would be low-risk individuals. Consequently, screening would have less benefit and there would be a higher degree of overdiagnosis.

Screening is a complex issue with many unsuspected variables playing an important role in the outcomes. Furthermore, a screening program would have to go through the rigorous control of a well-designed randomized controlled trial showing its effectiveness before it is put into practice.

Lastly, the best thing to do towards reducing healthcare inequity, in terms of healthcare policy, is to promote a universal coordinated public healthcare system,^4^ strongly based on primary care and without great interference from the private sector. Outside of such a system, promotion of any intervention will inexorably lead to more inequity.
